# Whose responsibility? Part 2 of 2: views of patients, families, and clinicians about responsibilities for addressing the needs of persons with mental health problems in Chennai, India and Montreal, Canada

**DOI:** 10.1186/s13033-021-00511-w

**Published:** 2022-01-10

**Authors:** Srividya N. Iyer, Ashok Malla, Megan Pope, Sally Mustafa, Greeshma Mohan, Thara Rangaswamy, Norbert Schmitz, Ridha Joober, Jai Shah, Howard C. Margolese, Padmavati Ramachandran

**Affiliations:** 1 Department of Psychiatry, McGill University, Montreal, Canada; 2 Prevention and Early Intervention Program for Psychosis (PEPP-Montreal), Douglas Mental Health University Institute, Montreal, Canada; 3 Schizophrenia Research Foundation (SCARF), Chennai, India; 4 Department of Population-Based Medicine, Institute of Health Sciences, University Hospital Tuebingen, Tuebingen, Germany; 5 Prevention and Early Intervention Program for Psychosis, McGill University Health Centre (PEPP-MUHC), Montreal, Canada

**Keywords:** Responsibility, Mental health, Psychosis, Culture, Stakeholder participation, Low- and middle-income countries, Needs, Early intervention services, Government, Family

## Abstract

**Background:**

Individuals with mental health problems have many insufficiently met support needs. Across sociocultural contexts, various parties (e.g., governments, families, persons with mental health problems) assume responsibility for meeting these needs. However, key stakeholders' opinions of the relative responsibilities of these parties for meeting support needs remain largely unexplored. This is a critical knowledge gap, as these perceptions may influence policy and caregiving decisions.

**Methods:**

Patients with first-episode psychosis (n = 250), their family members (n = 228), and clinicians (n = 50) at two early intervention services in Chennai, India and Montreal, Canada were asked how much responsibility they thought the government versus persons with mental health problems; the government versus families; and families versus persons with mental health problems should bear for meeting seven support needs of persons with mental health problems (e.g., housing; help covering costs of substance use treatment; etc.). Two-way analyses of variance were conducted to examine differences in ratings of responsibility between sites (Chennai, Montreal); raters (patients, families, clinicians); and support needs.

**Results:**

Across sites and raters, governments were held most responsible for meeting each support need and all needs together. Montreal raters assigned more responsibility to the government than did Chennai raters. Compared to those in Montreal, Chennai raters assigned more responsibility to families versus persons with mental health problems, except for the costs of substance use treatment. Family raters across sites assigned more responsibility to governments than did patient raters, and more responsibility to families versus persons with mental health problems than did patient and clinician raters. At both sites, governments were assigned less responsibility for addressing housing- and school/work reintegration-related needs compared to other needs. In Chennai, the government was seen as most responsible for stigma reduction and least for covering substance use services.

**Conclusions:**

All stakeholders thought that governments should have substantial responsibility for meeting the needs of individuals with mental health problems, reinforcing calls for greater government investment in mental healthcare across contexts. The greater perceived responsibility of the government in Montreal and of families in Chennai may both reflect and influence differences in cultural norms and healthcare systems in India and Canada.

**Supplementary Information:**

The online version contains supplementary material available at 10.1186/s13033-021-00511-w.

## Background

Individuals with mental health problems have many support needs, such as for financial resources; accessing mental health and substance use services and covering their costs; starting or resuming work or school; and accessing safe, affordable housing [1, 2].

Although most governments address some of these needs, there are significant disparities in mental health expenditures and coverage between countries [3, 4]. There are also striking differences in the organization of mental health and allied health and social service systems across, and sometimes within, nations [5]. Globally, median expenditure on mental health stands at around 2% of total government health expenditure, while mental disorders account for 12% of total disability-adjusted life years and 35% of total years lived with disability [4, 6]. As a result, across jurisdictions, many needs of persons with mental illnesses are not met or are inadequately met [1, 2]. At a societal level, there is also a need to raise awareness about mental health problems and reduce stigma [2].

In addition to governments, many other stakeholders—families; communities, philanthropic and non-governmental organizations; the private sector (e.g., psychiatrists in private practice); and persons with mental health problems themselves—take on the responsibility for meeting mental healthcare needs [1, 7, 8]. Along with the structure and financing of healthcare systems, the distribution of roles and responsibilities among stakeholders may be influenced by various psychological, sociological, and illness factors, such as prevailing views regarding individual versus societal responsibility for health and needs provision; attributions of responsibility for mental illness and substance use problems; views about autonomy versus relatedness; norms about caregiving and the family role; values (e.g., recovery, agency, etc.) underpinning mental healthcare provision; and type, severity and phase of the illness [8, 9]. The perceptions of various stakeholders regarding who should hold how much responsibility for addressing the needs of persons with mental illnesses remains largely unexplored. This is a critical knowledge gap because such perceptions can significantly influence the design of mental health services and policy. On a day-to-day basis, stakeholders’ perceptions can shape how they enact their own roles and whether and what kinds of support they provide and expect, and thereby influence outcomes. For instance, an individual with mental illness who sees it as the government’s role to provide financial support via disability benefits may feel less compelled to find work and their families with similar views may be less likely to support them in finding work. Conversely, clinicians and families who see it as the responsibility of families to provide housing to their ill family members may be less likely to advocate for government-subsidized housing for persons with mental health problems. Stakeholders may also feel discouraged and invalidated when the support they expect from other stakeholders is not forthcoming.

The salience of stakeholder perceptions about responsibilities for mental healthcare became evident during pilot studies that we conducted in the planning of a large comparative study of the two-year course of first-episode psychosis in Chennai, India and Montreal, Canada. In focus groups with patients, their families, and clinicians at both sites, participants acknowledged the shared roles of multiple parties in the mental health context, and saw responsibilities in relative rather than absolute terms (e.g., families’ role versus that of patients in helping patients resume work/school). Given that differences in perceptions about stakeholder responsibilities across the two contexts and between respondent groups (patients, families, and clinicians) emerged as important and potentially underpinning differences in service utilization and outcomes, we chose this as an area of investigation in our main study [10, 11].

Accordingly, we developed and administered a novel measure, the Whose Responsibility Scale (WRS), to determine how patients, family members, and treatment providers think responsibility for meeting various support needs of persons with mental health problems should be apportioned among persons with mental health problems, families, and the government. Part 1 of this two-part report describes this tool and details its development and psychometric testing.

Acknowledging important differences in the Canadian and Indian healthcare systems, we hypothesized that, compared to their Chennai counterparts, respondents in Montreal would ascribe higher levels of responsibility to the government for addressing mental healthcare needs. Universal healthcare is federally mandated in Canada and about 70% of healthcare costs are borne by the public healthcare system [12]. In striking contrast, 80% of healthcare expenditure in India is for private care of which 82% is out-of-pocket. There is no universal healthcare in India, and the system is extremely fragmented, with the private sector, including for-profit entities and non-governmental organizations, playing central roles [13].

Our second hypothesis was that compared to those in Montreal, respondents in Chennai would assign higher levels of responsibility to families (versus patients) for addressing mental healthcare needs. In India, limited government-provided coverage and a dearth of services and specialists have resulted in families playing substantial roles in mental healthcare provision [14–16]. Furthermore, most Indians, including those with mental health problems, live with their families, and there is a strong emphasis on familial values and networks [14, 17–19]. While the number of nuclear families in urban contexts in India may be increasing, family sizes still tend to be larger on average than in Canada, with a sizeable proportion of joint families or multi-generation households [20].

## Methods

### Research setting

This study is part of a two-year longitudinal study of outcomes of first-episode psychosis in Chennai, India and Montreal, Canada [21, 22]. Chennai and Montreal are both large metropolises, with populations of 6 million and 3.2 million, respectively. Montreal is in Quebec, which is the only province in Canada whose sole official language is French [12]. Chennai is in Tamil Nadu in the south of India, where people predominantly speak Tamil, and has a literacy rate of over 90% [23].

In Montreal, two early intervention services (EIS) for psychosis called Prevention and Early Intervention Program(s) for Psychosis (PEPP-Montreal and PEPP-MUHC) participated in the study. The Montreal programs are part of Quebec’s public healthcare system and are responsible for addressing the needs of young people with first-episode psychosis in specified geographic catchment areas. The Chennai site is the first-episode psychosis program at the Schizophrenia Research Foundation (SCARF), a mental health-focused non-governmental organization and a World Health Organization collaborating centre. In partnership with PEPP, SCARF built its first-episode clinical and research program between 2006 and 2008.

The Montreal and Chennai programs follow international guidelines for early intervention for psychosis [24–26]. They have similar inclusion and exclusion criteria, and accept referrals from a wide variety of sources, including patients and families. The EIS in both contexts offer a two-year treatment program with a recovery orientation and similar core components, including case management; flexible use of the lowest effective doses of second-generation antipsychotics; family psychoeducation; individual family intervention; and cognitive-behavioural therapy when needed. These core components were adapted to local preferences for greater acceptability (e.g., eliciting and addressing culturally-driven attributions for psychosis during family psychoeducation in Chennai). Both programs provide their services free of charge. At the Montreal programs, medication costs are covered through Québec's pharmacare program or, for a smaller proportion of patients, through private (usually parental) medication insurance. At SCARF, medications are provided free of cost to those who cannot afford to buy them, and cost is an important consideration (along with effectiveness) in the choice of psychiatric medications prescribed.

All study procedures complied with the ethical standards of the relevant national and institutional committees on human subject research and with the Helsinki Declaration of 1975, as revised in 2008, and were approved by the Institutional Review Board at SCARF, Chennai, and the Research Ethics Board at McGill University, Montreal.

### Participants

The study sample comprised patients, family members, and clinicians at the EIS at both sites. The samples for this report were recruited from the larger study samples which comprised 168 and 165 patients, and 168 and 129 families in Chennai and Montreal, respectively [22].

To be included, patients had to have a first episode of affective or non-affective psychosis that was not solely substance-induced (though concurrent substance use was accepted); be between 16 and 35 years old; be fluent in Tamil or English in Chennai and French or English in Montreal; have an IQ above 70 (as determined by testing in Montreal and as presumed to be indicated by no diagnosis of intellectual disability in Chennai); have no or less than 30 days of previous treatment with antipsychotic medication; and not have a history of organic brain or neurodevelopmental disorders. Family members were patients’ parents, siblings, or partners/spouses, and clinicians were psychiatrists, case managers, and other allied healthcare professionals (e.g., employment support specialists, psychologists, etc.).

### Assessments

At both sites, assessments were administered by similarly trained research staff in English or French in Montreal, and English or Tamil in Chennai, depending on participants’ preferences. All measures described in this report were collected upon entry to the EIS (baseline). Sociodemographic information for patients, families, and clinicians was collected. Primary and co-morbid substance use diagnoses were established using the Structured Clinical Interview for DSM-IV-TR Axis I disorders (SCID-I) [27]. The Circumstances of Onset and Relapse Schedule (CORS) [28] was administered to estimate the duration of untreated psychosis (DUP) and age at onset of psychosis. Symptom severity was assessed with the Scale for the Assessment of Positive Symptoms (SAPS) [29] and the Scale for the Assessment of Negative Symptoms (SANS) [30]. As detailed in our previous publications [22, 31], acceptable levels of inter-rater reliability were achieved for these measures within and between the two sites.

#### Whose Responsibility Scale (WRS)

The WRS is a 22-item scale designed to assess respondents’ opinions of the responsibility that should be borne by three stakeholders (the government, persons with mental health problems, and their families) in addressing each of seven support needs of persons with mental health problems (general financial support, housing support, reintegration into education and/or employment, costs of mental health services, medications, and substance use treatment programs, and stigma reduction/awareness).

The WRS items were modeled after an item from the World Values Survey [32] rated on a visual analogue scale from 1 to 10, where scores closer to 1 indicate greater agreement with the view that governments should take more responsibility to ensure that everyone is provided for, and scores closer to 10 indicate greater agreement with the view that people should be more responsible for providing for themselves. This item is widely used as an indicator of an individual’s political orientation [33, 34].

In the WRS, for each of the seven support needs, a set of three items are presented, with the first requiring respondents to contrast the role of the government versus that of persons with mental health problems; the second contrasting the role of the government versus that of families of persons with mental health problems; and the third contrasting the role of families versus that of persons with mental health problems, resulting in 21 items. We also administered the original item from the World Values Survey, for a total of 22 items. On all items, 1 indicates complete agreement with the statement on the left, 10 indicates complete agreement with the statement on the right, and selection of any other number in between reflects one’s relative weighting of the responsibility of each party. For items comparing the role of the government versus persons with mental health problems, lower scores indicate a higher level of responsibility being assigned to the government for meeting the need in question. For items comparing the role of the government versus families, lower scores similarly indicate a higher level of responsibility being assigned to the government. Finally, for items comparing the role of families versus persons with mental health problems, lower scores indicate a higher level of responsibility being assigned to families for meeting the need in question.

Item scores were summed up and then averaged to arrive at three composite scores indicating the extent of overall responsibility that respondents felt should be assigned to (1) governments versus persons with mental health problems; (2) governments versus families; and (3) families versus persons with mental health problems, across all support needs. Scores were also derived separately for each of the seven support needs to enable a comparison of responsibilities assigned to each stakeholder pair by the need area rated.

The WRS was tested and found to have acceptable test–retest reliability and internal consistency across sites (Chennai and Montreal); languages (English, French and Tamil); and rater groups (patients, families, and clinicians) (see Part 1 of this report for details).

### Statistical analysis

Data were analysed using SPSS version 24. Group comparisons on demographic and clinical characteristics were made using unpaired *t* and χ^2^ tests for continuous and categorical variables, respectively, with two-tailed *p* < 0.05 level of significance. The responsibility for support needs ascribed to different stakeholder pairs is presented as means (ranging from 1–10) and standard deviations, and individual item or composite scores are used for our comparative analyses. This statistical approach aligns with the one taken in research using the World Values Survey item that we included in the WRS [33, 34].

As main analyses, three two-way analyses of variance (ANOVAs) were conducted to examine the effects of site (Chennai vs. Montreal), rater (patient vs. family vs. clinician), and their interaction on relative responsibilities assigned to government versus persons with mental health problems; government versus families; and families versus persons with mental health problems. Ratings on the World Values Survey item were used as a covariate when examining responsibilities assigned to the government versus other stakeholders, because respondents’ views on the government’s responsibility for providing for people can influence their opinions about the government’s ideal role in health care provision [35, 36]. In addition, a two-way ANOVA was conducted to examine site, rater, and interaction effects on this standalone item from the World Values Survey.

Post-hoc pairwise comparisons with Bonferroni adjustment for multiple comparisons were conducted and effect sizes (partial eta squared; ƞp^2^) were computed. Effect sizes were categorized as small (ƞp^2^ = 0.01), medium (ƞp^2^ = 0.06) or large (ƞp^2^ = 0.14) [37].

To understand opinions about responsibility for each support need, we conducted separate two-way ANOVAs for each of the seven needs. In addition, we used one-way ANOVAs to examine whether responsibility assigned to the government varied by area of need.

## Results

### Sample characteristics (Table 1)

**Table 1:** Characteristics of respondents at baseline

	Patients			
	Montreal N = 86	Chennai N = 164	t (df)	P
	Mean (SD)	Mean (SD)		
Continuous variables				
Age at onset of current episode of psychosis	23.88 (5.41)	25.84 (5.21)	2.75 (241)	**0.006**
Years of education	12.54 (2.60)	11.84 (3.91)	1.67 (229)	0.097
SAPS total severity score	34.16 (15.28)	20.12 (9.79)	7.53 (114)	** < 0.001**
SANS total severity score	23.55 (13.41)	21.39 (15.63)	1.11 (194)	0.267
Log DUP	1.14 (0.75)	1.08 (0.60)	0.59 (124)	0.558
DUP median (range) in weeks	11.0 (0.0–684.3)	11.6 (0.3–223.0)	NA	NA

	N (valid %)	N (valid %)	Χ^2^ (df)	P
Categorical variables				
Men	52 (60.5%)	82 (50.0%)	4.72 (2)	0.095
Single	79 (92.9%)	105 (64.0%)	24.27 (1)	** < 0.001**
SCID-IV diagnosis of schizophrenia-spectrum disorder	56 (65.1%)	146 (90.1%)	23.25 (1)	** < 0.001**
SCID-IV diagnosis of substance abuse/dependence	29 (38.2%)	17 (10.5%)	25.39 (1)	** < 0.001**

	Families			
	Montreal N = 64	Chennai N = 164	Χ^2^ (df)	P
	N (valid %)	N (valid %)		
Relation to patient				
Parent*	48 (80.0%)	87 (53.0%)	14.85 (3)	**0.002**
Spouse*	5 (8.3%)	45 (27.4%)		
Sibling	6 (10.0%)	21 (12.8%)		
Other	1 (1.7%)	11 (6.7%)		
Women	47 (78.3%)	79 (48.2)	16.24 (1)	** < 0.001**
Age group (years)			37.24 (5)	** < 0.001**
21–30	6 (10.3%)	11 (6.7%)		
31–40*	5 (8.6%)	56 (34.1%)		
41–50	9 (15.5%)	30 (18.3%)		
51–60	26 (44.8%)	65 (39.6%)		
61–70*	11 (19.0%)	2 (1.2%)		
71–80	1 (1.7%)	0 (0.0%)		
Education			47.41 (5)	** < 0.001**
Less than high school	1 (1.7%)	9 (5.5%)		
High school*	9 (15.3%)	100 (61.0%)		
College/vocational degree*	19 (32.2%)	25 (15.2%)		
Bachelor's*	23 (39.0%)	17 (10.4%)		
Master's	5 (8.5%)	11 (6.7%)		
Doctorate	2 (3.4%)	2 (1.2%)		

	Clinicians			
	Montreal N = 29	Chennai N = 21	Χ^2^ (df)	P
	N (valid %)	N (valid %)		
Women	17 (58.6%)	19 (90.5%)	6.13 (1)	**0.013**
Age group (years)				
21–30*	1 (4.5%)	16 (76.2%)	24.60 (3)	** < 0.001**
31–40	7 (31.8%)	2 (9.5%)		
41–50	7 (31.8%)	3 (14.3%)		
51–60*	7 (31.8%)	0 (0.0%)		
Profession			14.07 (2)	** < 0.001**
Psychiatrist	7 (24.1%)	1 (4.8%)		
Case manager	13 (44.8%)	20 (95.2%)		
Allied health care professionals	9 (31.0%)	0 (0.0%)		

*DUP* Duration of untreated psychosis, *SAPS* Scale for the Assessment of Positive Symptoms, *SANS* Scale for the Assessment of Negative Symptoms, *SCID IV* Structured Clinical Interview for DSM-IV

Bold indicates significant differences between Montreal and Chennai

^*^Indicates significant post-hoc differences between Montreal and Chennai

The sample comprised 164 patients, 164 family members, and 21 clinicians in Chennai; and 86 patients, 64 family members, and 29 clinicians in Montreal. Patients in Montreal and Chennai were comparable on gender distribution, years of education, negative symptom severity and DUP. Montreal patients had a lower age at onset, more severe positive symptoms, and were more likely to be single and have co-morbid substance use than patients in Chennai. Compared to their Montreal counterparts, Chennai patients were more likely to have a primary diagnosis of a schizophrenia-spectrum disorder rather than an affective psychosis disorder.

Montreal family raters were predominantly parents, whereas the Chennai sample also included spouses. Family members also tended to be older and more educated in Montreal.

Compared to Montreal, the Chennai sample of clinicians included fewer psychiatrists, more women, and tended to be younger.

### Respondents vs. non-respondents

A quarter of the patients from the larger study sample (24.9%, 83/333) did not respond to the WRS at baseline. Only four non-respondents were from Chennai. Given that the majority of the non-respondents were from Montreal (n = 79/83; 95.2%), we compared respondents vs. non-respondents in Montreal. Respondents and non-respondents in Montreal were comparable on age at entry to the EIS, age at onset of psychosis, gender, marital status, education, employment, DUP, primary diagnosis, substance use, and baseline positive and negative symptom severity.

A quarter of families (23.2%, 69/297) did not respond to the WRS at baseline, with the majority (65/69) being from Montreal. A comparison of respondent and non-respondent Montreal families revealed no significant differences in age, gender, education, or relationship to the patient (e.g., parent versus sibling).

### Main and interaction effects for overall responsibility for addressing needs (Table 2; Fig. 1a–c)

**Table 2:** Comparisons of overall responsibility assigned to stakeholder pairs for overall support

	Patients		Families		Clinicians		Statistics
	Montreal N = 85 M (SD)	Chennai N = 164 M (SD)	Montreal N = 63 M (SD)	Chennai N = 164 M (SD)	Montreal N = 29 M (SD)	Chennai N = 21 M (SD)	F(df), p, η_p_^2^
Government vs Persons with mental health problems	3.98 (2.21)	4.33 (1.88)	2.73 (1.52)	3.55 (1.57)	3.55 (1.61)	3.95 (1.23)	**Site effect** F = 16.96 (1,519), < **0.001**; η_p_^2 =^ 0.032 **Rater effect** ^a^ F = 11.41 (2,519), < **0.001**; η_p_^2 =^ 0.042 Interaction effect F = 0.23 (2,519), 0.797; η_p_^2 =^ 0.001
Government vs Families	4.36 (2.21)	5.24 (2.17)	3.43 (1.63)	4.81 (1.82)	3.78 (1.54)	4.61 (0.89)	**Site effect** F = 41.43 (1,519), < **0.001**; η_p_^2 =^ 0.074 **Rater effect** ^b^ F = 3.06 (2,519), **0.048**; η_p_^2 =^ 0.012 Interaction effect F = 0.37 (2,519), 0.689; η_p_^2 =^ 0.001
Families vs Persons with mental health problems	5.17 (1.76)	4.31 (1.84)	4.02 (1.36)	3.65 (1.53)	4.97 (1.18)	4.33 (0.89)	**Site effect** F = 10.93 (1,521), < **0.001**; η_p_^2 =^ 0.021 **Rater effect** ^c^ F = 16.51 (2,521), < **0.001**; η_p_^2 =^ 0.060 Interaction effect F = 1.16 (2,521), 0.315; η_p_^2 =^ 0.004

Significant differences, p < 0.05, are bold.* Vs.* versus

Post-hoc pairwise comparisons with Bonferroni adjustment: ^a^ Family raters attributed significantly more responsibility to government than patient raters, p < 0.001; ^b^ Family raters attributed more responsibility to government than patient raters, but this did not reach statistical significance, p = 0.074; ^c^ Family raters attributed significantly more responsibility to families than both patient raters, p < 0.001 and clinician raters, p = 0.006

**Fig. 1:**
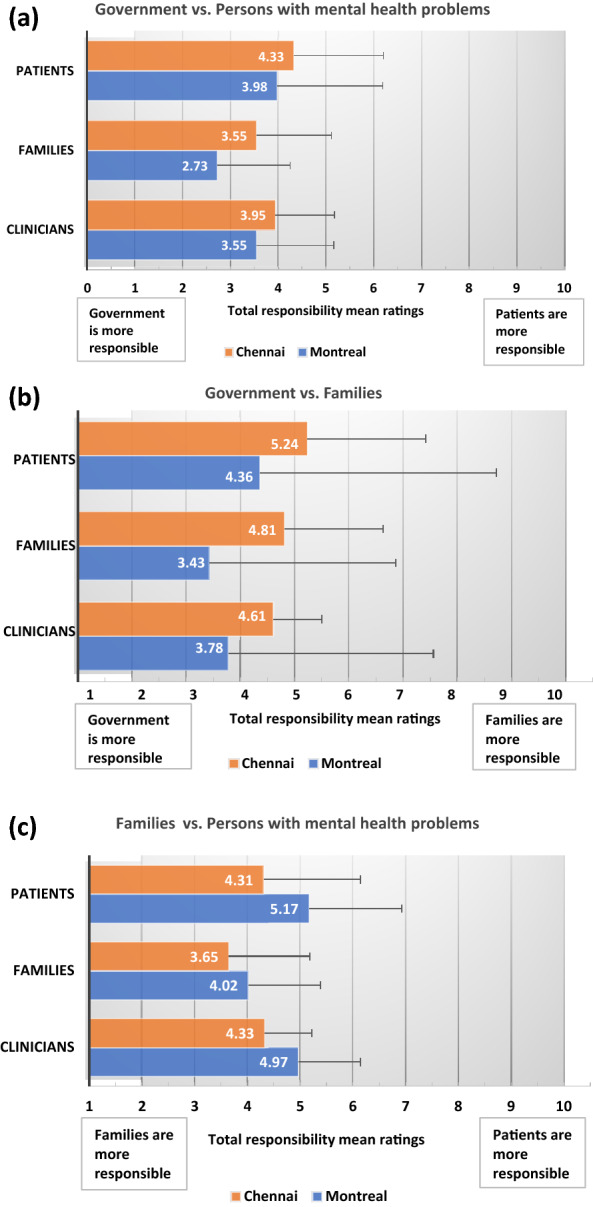
**a** Total responsibility assigned to government versus persons with mental health problems (patients). Data are presented as means and standard deviations. The lower the score, the higher the responsibility assigned to the government. Montreal raters assigned significantly higher total responsibility to governments compared to Chennai raters. Family respondents assigned significantly higher total responsibility to governments than did patient respondents. **b** Total responsibility assigned to government versus families. Data are presented as means and standard deviations. The lower the score, the higher the responsibility assigned to governments. Montreal raters assigned significantly higher total responsibility to government than Chennai raters. Family respondents assigned significantly higher total responsibility to governments than did patient respondents. **c** Total responsibility assigned to families versus persons with mental health problems (patients). Data are presented as means and standard deviations. The lower the score, the higher the responsibility assigned to families. Chennai raters assigned significantly higher total responsibility to families than Montreal raters. Family member respondents assigned significantly higher total responsibility to families than did patient and clinician respondents

#### Role of government versus persons with mental health problems

Across both sites, all raters held governments more responsible than persons with mental health problems (means were always below 5, with 1 indicating greater government responsibility) for addressing the various needs of individuals with mental health problems, taken together. After controlling for the World Values Survey item, two-way ANOVA simple main effects showed that, as hypothesized, raters in Montreal assigned significantly more responsibility to the government than did raters in Chennai (small effect size). With respect to rater effects, family members assigned significantly more responsibility to the government than to persons with mental health problems, compared to patients who responded to the survey (small effect size). The interaction effect was not significant.

#### Role of government versus families

The same pattern was observed with respect to responsibility assigned to government versus family, with significant site and rater effects, and the interaction effect not being significant. As predicted, compared to raters in Chennai, those in Montreal assigned significantly more responsibility to the government versus families for addressing the various needs of persons with mental health problems (medium effect size). Again, family raters assigned significantly more responsibility to the government than did patients who responded to the survey (small effect size).

#### Role of families versus persons with mental health problems

With respect to the assignment of responsibility to families versus persons with mental health problems, as hypothesized, Chennai raters assigned significantly higher levels of responsibility to families than did Montreal raters (small effect size). There was also a significant rater effect (medium effect size), with family raters, irrespective of site, assigning more responsibility to families (versus persons with mental health problems) for addressing the needs of individuals with mental health problems, than did patient and clinician raters. Again, the interaction effect was not significant.

### Item from the World Values Survey

All raters at both sites assigned more responsibility to governments than people themselves for ensuring that everyone is generally provided for. A two-way ANOVA showed a significant site effect [Montreal: Mean = 4.81, SD = 2.67, Chennai: Mean = 3.89, SD = 2.66, F (1, 520) = 5.69, p = 0.017, η_p_^2^ = 0.011]; rater effect [Patients: Mean = 4.49, SD = 2.93, Families: Mean = 3.87, SD = 2.48, Clinicians: Mean = 4.31, SD = 2.24; F (2, 520) = 5.04, p = 0.007, η_p_^2^ = 0.019] and site-rater interaction effect [F (2, 520) = 3.85, p = 0.022, η_p_^2^ = 0.015] on responses to the World Values Survey item. All effect sizes were small. Chennai raters attributed significantly more responsibility to the government (versus people themselves) for generally providing for people than did Montreal raters.

**Fig. 2:**
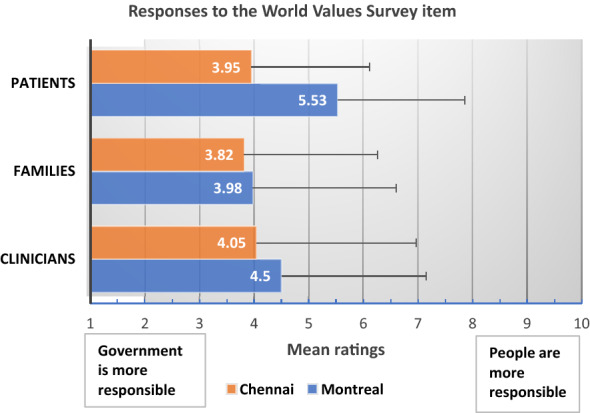
Responses to the World Values Survey item assigning responsibility to government versus people for needs provision. Data are presented as means and standard deviations. The lower the rating, the higher the responsibility assigned to the government. Site: Chennai raters assigned more responsibility to government than Montreal raters; Raters: Family raters assigned more responsibility to government than patients; Site x Rater interaction: Patients in Montreal assigned significantly less responsibility to government than patients in Chennai

With respect to the rater effect, pairwise comparisons showed that family raters assigned more responsibility to the government for providing for people than did patient raters (p = 0.005). Pairwise comparisons in relation to the significant interaction effect showed that patient raters in Chennai (Mean = 3.95, SE = 0.21) assigned significantly more responsibility to the government for providing for people than did patient raters in Montreal (Mean = 5.53, SE = 0.29; p < 0.001, Fig. 2).

### Secondary analyses: who is responsible for each of the seven support needs? (Additional files 1 and 2)

The pattern of findings for relative responsibilities for each support need was generally similar to that of total responsibility for all needs combined, in that governments were seen as more responsible for addressing most needs than were persons with mental health problems or families, with means generally falling ≤ 5.

There were consistent patterns of similarities and differences between Chennai and Montreal in responsibility ratings for each of the support needs with a few notable exceptions:

- Governments versus persons with mental health problems: Chennai and Montreal raters did not differ in the level of responsibility they assigned to governments versus persons with mental health problems for meeting needs relating to general financial support, reintegration into education and/or employment, covering the costs of mental health services or medications, and stigma reduction/awareness. Compared to their Chennai counterparts, Montreal raters did, however, assign greater responsibility to the government versus persons with mental health problems for meeting needs related to housing and costs of substance use treatment. The latter also showed a significant site-rater interaction effect, with Montreal patient and family raters assigning significantly higher responsibility to the government for covering the costs of substance use treatment than their Chennai counterparts.
- Governments versus families: Both Chennai and Montreal raters ascribed equal and high levels of responsibility to the government (versus families) for addressing general financial support needs and stigma reduction/awareness. On the other hand, compared to Chennai raters, those in Montreal assigned higher responsibility to the government versus families for meeting needs related to housing, reintegration into education and/or employment, and covering the costs of medications, substance use treatment, and mental health services. The latter also showed a significant site-rater interaction effect, with Montreal family raters assigning significantly higher responsibility to the government for covering the costs of mental health services than their Chennai counterparts.
- Families versus persons with mental health problems: Compared to Montreal raters, Chennai raters consistently ascribed more responsibility to families versus persons with mental health problems for meeting each of the seven support needs, with one notable exception. When it came to covering the costs of substance use treatment, Chennai raters held families responsible to a comparatively lower degree (versus persons with mental health problems) than did their Montreal counterparts. This was in striking contrast to their opinion about every other need and all needs taken together, with Chennai raters always ascribing more responsibility to families than did raters in Montreal, as had been hypothesized.

With respect to similarities and differences in responsibility ratings assigned for each of these seven support needs by patient, family, and clinician raters (see Additional file 2), we found that:

- Family raters: For each of the seven needs and at both sites, family raters assigned more responsibility to the government versus persons with mental health problems than did patient raters. Family raters also allocated significantly more responsibility to the government versus persons with mental health problems than did clinician raters for addressing the financial needs of persons with mental health problems and for stigma reduction/awareness. Similarly, family raters assigned more responsibility to the government versus families than did patient raters for meeting four needs (housing, reintegration into school/work, substance use treatment costs, and stigma reduction). Notably, when it came to assigning responsibility to families versus persons with mental health problems, family raters ascribed more responsibility to families than did clinicians for meetings three specific need areas: financial support, reintegration into education and employment, and covering the costs of mental health services.
- Clinician raters: Clinician raters assigned significantly more responsibility to the government versus persons with mental health problems and families for covering the costs of substance use treatment than did patient raters. Similarly, clinicians assigned more responsibility to the government versus families for covering the costs of mental health services.

### Exploratory analyses

Given the relatively high levels of responsibility assigned to the government versus persons with mental health problems for addressing support needs, we conducted post-hoc analyses to examine whether the responsibility assigned to the government varied by support need. These post-hoc analyses were conducted separately for Montreal and Chennai, combining ratings from patient, clinician, and family raters (post-hoc pairwise comparisons are presented in Additional file 3).

**Fig. 3:**
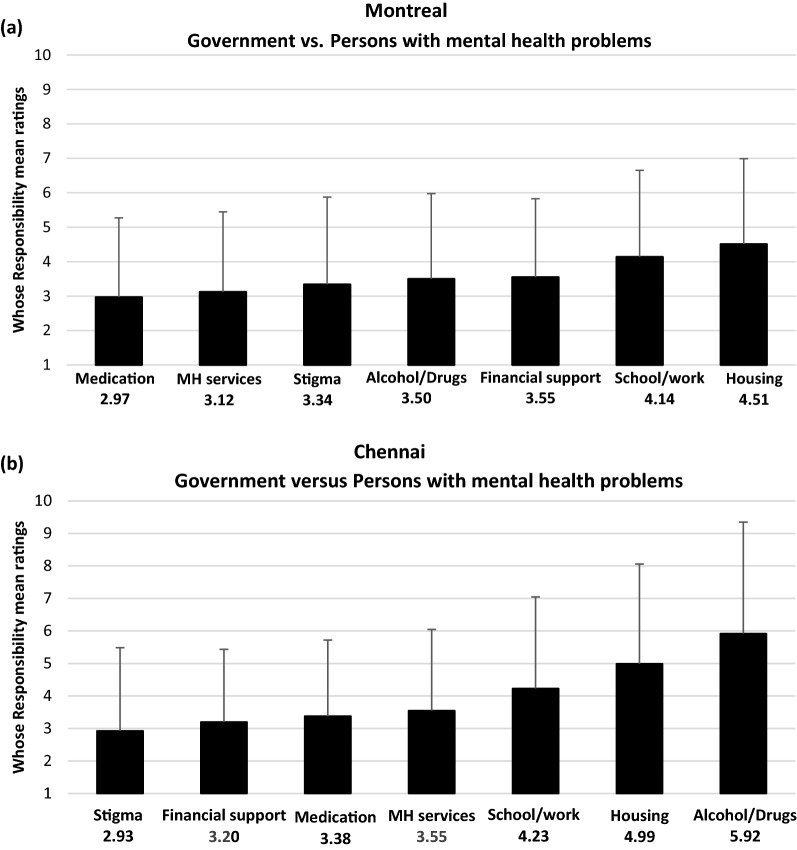
**a** Responsibility assigned to government versus persons with mental health problems by need: Montreal raters (MH = mental health). Data are presented as means and standard deviations. The lower the mean rating, the higher the responsibility assigned to the government. As you go from left to right, responsibility assigned to government is lower. Government was assigned significantly less responsibility for housing than all other need areas, except work/school. Government was assigned significantly less responsibility for work/school integration than for costs of mental health services, medication and stigma. **b** Responsibility assigned to government versus persons with mental health problems by need: Chennai raters (MH = mental health). Data are presented as means and standard deviations. The lower the mean rating, the higher the responsibility assigned to the government. As you go from left to right, responsibility assigned to government is lower. Government was assigned significantly less responsibility for needs related to alcohol/drugs treatment, housing and work/school than for all other need areas

In Montreal, in descending order of level of responsibility assigned to the government versus persons with mental health problems, the seven support needs were: covering the costs of medication, covering the costs of mental health services, stigma reduction, covering the costs of substance use treatment, general financial support, help reintegrating into school/work, and housing support (Fig. 3a). In Chennai, in descending order of level of responsibility assigned to the government, the seven support needs were stigma reduction, general financial support, covering the costs of medication, covering the costs of mental health services, help reintegrating into school/work, housing support, and covering the costs of substance use treatment (Fig. 3b).

At both sites, governments were assigned relatively less responsibility for addressing needs related to housing and school/work reintegration compared to other support needs. In Chennai, the government was ascribed less responsibility for covering the costs of substance use treatment than for addressing all other needs. Chennai raters also seemed to hold the government more responsible for stigma reduction and awareness than for housing, school/work integration, and cost of mental health and substance use services.

## Discussion

Using a purpose-built tool, we examined how patients, families, and clinicians in two distinct sociocultural contexts ascribed relative responsibility to governments, patients, and their families for meeting the needs of persons with mental health problems. As expected, Montreal raters ascribed higher levels of overall responsibility to the government than their Chennai counterparts, and Chennai raters ascribed higher levels of overall responsibility to families than Montreal raters.

### Responsibility of governments

At both sites, all respondents—patients, families, and clinicians—held governments responsible to a substantial degree for addressing the support needs of individuals with mental health problems. This aligns with the growing demand in high- and low-and middle-income countries alike for governments to make stronger policy and funding commitments to mental health [6]. Respondents’ experiences of living with a serious mental health problem like psychosis or caring for a person with psychosis may have increased their awareness of needs and of the important role of governments in addressing them. Future research in general populations using the WRS can help establish whether these views are commonplace or products of experiences with psychosis.

Citizens’ expectations of their government’s role and responsibilities are influenced by their knowledge of, and experiences in, their current healthcare systems. There are important differences between the Canadian and Indian healthcare systems [13]. Canadians have access to universal healthcare under the Canada Health Act of 1984 [5]. About 70% of health costs in Canada are met through public funding, with the remaining 30% (including, e.g., medication, dentistry, etc.) being covered by private insurance and out-of-pocket expenses [12]. Philosophically, India too emphasizes “health for all” through its National Health Policy of 1983 [38]. However, it does not have universal healthcare, and private care expenses account for roughly 80% of total healthcare spending. In this multi-tiered healthcare system with public, for-profit, and non-governmental players, the quality of healthcare is perceived to be better in private/for-profit services than in public services [13, 39]. It is therefore unsurprising that Canadian respondents consistently ascribed higher levels of responsibility to the government for addressing mental healthcare needs than Indian respondents. This was especially true when comparing the responsibility of governments versus families (medium effect size).

Irrespective of site, families felt that the government should be held more responsible for addressing needs than did patients. As a group, families tended to include more women and older individuals, and some previous research has shown these very groups to be more supportive of welfare state policies [40, 41]. Furthermore, family members’ caregiving experiences and associated strain may shape their perceptions about governments’ responsibility.

Understandably, perceptions regarding government responsibilities often varied by type of support need, and context. At both sites, the level of responsibility assigned to the government for addressing housing needs was significantly lower than that for addressing other needs, with Chennai raters (compared to Montreal raters) ascribing less responsibility to the government for housing needs. This may be because most patients in Chennai lived with their families. This is generally the case in India because of cultural norms and economic constraints [18, 20]. Furthermore, government-supported or subsidized housing for individuals with mental illnesses and other disabilities and low-income housing are virtually non-existent in India [42–46]. In Canada, supported or subsidized housing options are considered part of community care for serious mental disorders, even if their availability is limited compared to demand [47]. EIS’ value normalization, recovery, and community living. Having brought into this philosophy, our Montreal respondents may have seen housing support as a less important priority for governments than other need areas. Moreover, many young patients even in the Montreal cohort lived with their families at the time of entry into treatment. However, these views about government responsibility for addressing housing needs held by our respondents may evolve over the course of treatment and may be substantially different from those held by specific subgroups such as homeless youth with psychosis [48, 49].

Chennai raters ascribed less responsibility to the government for covering the costs of substance use treatment than for addressing all other needs (except housing), and deemed governments less responsible for this need than did Montreal raters. Most strikingly, this was the only need area for which Chennai raters held families less responsible than did Montreal raters. Our previous critical literature review [8] showed that attributions about the causes and controllability of mental illnesses may influence perceptions of responsibility. There is evidence that attributions and views about substance use problems may differ from those about mental illnesses [50, 51]. Individuals are less likely to allocate resources to supporting people deemed personally responsible for their problems [52]. This may explain Chennai respondents’ views about substance use problems [50, 51]. Such views about personal responsibility and the controllability of substance use may be more widespread in contexts with lower frequency of substance use problems as in Chennai. Prior research has shown that causal and controllability attributions influence perceptions about responsibility for mental illness and on stigma [52–54]. However, whether this extends to perceptions of responsibility for addressing the needs of persons with mental health and substance abuse problems needs to be explored.

Our findings also suggest that individuals’ views on governments’ responsibility for mental healthcare needs may be related to, and yet distinct from, their views on governments’ responsibility for people generally. Montreal raters held the government slightly less responsible for ensuring that general needs are provided for than did their Chennai counterparts. However, when it came to mental healthcare needs, they ascribed significantly higher levels of responsibility to the government than did Chennai raters. Healthcare needs may be seen as more clearly falling under the ambit of government, particularly in societies with a public healthcare system. Also, respondents in high-income countries with relatively higher standards of living may not perceive themselves to be as needy of general government support as those in low-and middle-income countries.

### Responsibility of families

As hypothesized, Chennai raters consistently ascribed more responsibility to families for addressing the needs of persons with mental health problems than did Montreal raters. In India, a paucity of services and specialists has meant that families bear, and are ascribed, much of the responsibility for mental healthcare [14]. Familial structures, values, and living arrangements also contribute to families being more involved in addressing the needs of persons with mental health problems in India [15, 16, 19]. In our sample, Chennai families had more contact with their loved one’s clinical team throughout treatment than did Montreal families [21]. This high level of family involvement may have resulted in Chennai raters ascribing higher levels of responsibility to families. Conversely, it may also be true that families play a larger role in mental healthcare when they and other stakeholders see their roles and responsibilities as significant. Indeed, research conducted in Western countries suggests that many families would play a larger role in their loved ones’ care if given the opportunity [55–57]. This is important, because there is a wealth of evidence for the positive impacts of family support and involvement on mental health outcomes [58], including in psychosis [59–63]. The stronger emphasis on the individual patient and on consent, privacy and confidentiality considerations in contexts like Canada, compared to India, may contribute to stakeholders ascribing lower levels of responsibility to families and, as a corollary, families playing a more limited role in these contexts [16, 17, 64].

Our study is novel in documenting that perceptions about the role of the family vary not only by geo-cultural context, but also by the stakeholder group that a rater belongs to. Irrespective of context, family raters assigned more responsibility to families for addressing all needs taken together for each of the seven support needs than did patient and clinician raters, and rated family responsibility as higher compared to patient raters. While clinicians and patients value the family’s role and presence, they may also perceive a need to balance families’ roles and responsibilities with those of patients, with a view to ultimately fostering patient independence and recovery. Our previous qualitative research [9] also revealed that some patients were reluctant to assign too much responsibility to families for fear of overburdening them. These divergent views between patients, clinicians, and family members may contribute to families feeling excluded and less involved in their loved ones’ care than they desire, particularly in Western contexts like Montreal [55–57, 65].

### Responsibility of patients

All raters across sites assigned relatively less responsibility to persons with mental health problems than they did to governments and families. This may be a function of the focus of the WRS on larger macro-level needs (such as employment and housing services, stigma reduction, etc.). These findings, however, must not be taken as evidence against the valuing of patients’ agency. It is possible for people to opine that larger social structures (especially governments) must provide needed services and resources, while at the same time believing that persons with mental health problems should exercise agency in using them. This resonates with the literature on the social determinants of health [66], responsibility for physical health [67, 68], recovery [69, 70], and responsibility in mental health [9], which all highlight the need to situate individual responsibility and agency within a larger context where social structures are responsible for creating conditions that enable individual agency and responsibility. Furthermore, that Montreal raters, compared to Chennai raters, ascribed less responsibility to families than to patients suggests that sociocultural contexts shape views regarding the interplay between agency and structures.

Interestingly, compared to their families, patients ascribed lower levels of responsibility to governments and families (versus persons with mental health problems). Together, these findings suggest that patients value their own responsibility for meeting their support needs more than other stakeholders do, in line with our previous qualitative findings [9]. Given this emphasis on self-reliance, patients can experience self-blame and frustration when they cannot needs like healthcare that are or should rightly be in the purview of governments.

### Strengths and limitations

Our study’s chief strength is its novel focus on patients’, families’, and clinicians’ attributions of the relative responsibilities of patients, governments, and families for meeting the support needs of individuals with mental health problems in two distinct sociocultural contexts. This construct is supported by evidence and theory as was shown in our critical review [8]. Our findings also strengthen the case for capturing the perspectives of diverse stakeholders in health services research and yield new avenues for inquiry, such as enlisting stakeholder perceptions in setting policy priorities and examining the impact of perceptions regarding responsibility on the actual involvement of varied stakeholders in mental healthcare provision.

However, our study also has some limitations. The sample sizes were uneven at the two sites, with much greater participation among patients and families in Chennai than in Montreal. As those who did not complete the WRS were no different on any of the tested characteristics from those who completed the measure, we could not ascertain what may have differentiated respondents from non-respondents in Montreal. Nonetheless, the patterns of our study’s findings were consistent and in the hypothesized directions. Yet, it is possible that our findings in Montreal are not representative and may better reflect the views of individuals with higher levels of engagement in services and research.

Other support needs (e.g., coverage for psychotherapy) may have been important to assess. This notwithstanding, our measure is broad in its inclusion of seven key need areas that were based on previous literature [1, 71–77] and our qualitative work [9–11].

While our measure focused on the roles of governments, families, and persons with mental health problems, other systems (e.g., schools) also contribute to mental healthcare provision. Therefore, the clarification of their mandates versus those of other parties may be beneficial [78].

This study examined responsibility perceptions only at the point of entry into treatment. However, such perceptions may shift over time [79]. Finally, our study also presents perspectives from only one clinical context each in Canada and India. Future research using the WRS in the general population, in populations with other mental health problems across varied geo-cultural contexts, and in other settings (e.g., community peer support organizations) is warranted.

## Conclusion

An important implication of our findings is that it would be desirable for healthcare systems to equilibrate the assignment of responsibilities for meeting various mental healthcare needs to different stakeholder groups in a manner that is responsive to local sociocultural and economic realities. In the service context, views regarding one’s own and others’ ideal responsibilities may shape individuals’ and groups’ expectations, roles, levels of participation and (dis)satisfaction [65]. Such views can be explored through open and respectful dialogue. Insights from locally conducted research using tools such as the WRS can support this dialogue by serving as a frame of reference for stakeholders desiring to understand their responsibility views relative to others in their context. Crucially, such research can also be a much-needed means by which stakeholder views can inform mental health service and policy design and reform, and shed light on the iterative ways in which structures and contexts and expectations about roles and responsibilities shape one another.

Our respondents echo the growing calls for governments to provide more systematically and universally for mental healthcare [2, 80, 81]. It is important, however, that governments not only heed this call, but do so in manners that recognize and leverage the roles that other stakeholders play and can play in supporting persons with mental health problems. Ultimately, the best-functioning systems may be those that support and facilitate patient agency and familial involvement, within a larger overall context of governments providing comprehensive mental health services and additional supports for those needing them (e.g., housing) [9, 67, 68].

## Supplementary Information

**Additional file 1**. Comparisons of responsibility assigned to stakeholder pairs by type of support.

**Additional file 2**. Post-hoc tests for significant differences between raters (refer to additional file 1).

**Additional file 3**. Post-hoc tests for significant differences between support needs in Montreal and Chennai (refer to Fig. 3).

## Data Availability

The measure reported on in this paper, Whose Responsibility Scale, is included as Supporting Information in Part 1 of the report. To use or modify the scale, please contact its primary creator at srividya.iyer@mcgill.ca. The datasets generated and/or analysed during the current study are not publicly available due to ethics approval from participants not covering public sharing but are available from the corresponding author on reasonable request.

## Abbreviations

DUP: Duration of untreated psychosis
EIS: Early Intervention Service
PEPP: Prevention and Early Intervention Program for Psychosis
SCARF: Schizophrenia Research Foundation
WRS: Whose Responsibility Scale
Vs: Versus
