# Supplementary material for: Whose responsibility? Part 2 of 2: views of patients, families, and clinicians about responsibilities for addressing the needs of persons with mental health problems in Chennai, India and Montreal, Canada
Source: Int J Ment Health Syst. 2022 Jan 10;16:2. doi: 10.1186/s13033-021-00511-w (PMC8744303; doi:10.1186/s13033-021-00511-w)
Supplement: Supplementary file 1 — Additional file 1. Comparisons of responsibility assigned to stakeholder pairs by type of support. [file 13033_2021_511_MOESM1_ESM.docx]

**Additional file 1. Comparisons of responsibility assigned to stakeholder pairs by type of support.**

1. **General financial support**

|  | **PATIENTS** | | **FAMILIES** | | **CLINICIANS** | | **STATISTICS** |
| --- | --- | --- | --- | --- | --- | --- | --- |
|  | **Montreal** EMM (SE) | **Chennai**  EMM (SE) | **Montreal**  EMM (SE) | **Chennai**  EMM (SE) | **Montreal**  EMM (SE) | **Chennai**  EMM (SE) | **F, p, η_p_^2^** |
| **Government vs.  Persons with mental health problems** | 3.73 (0.24) | 3.36 (0.17) | 3.06 (0.28) | 2.94 (0.17) | 4.12 (0.42) | 4.00 (0.49) | Site effect  F= 0.61, 0.434;  η_p_^2^ = 0.001  **Rater effect**  F= 5.51, **0.004**;  η_p_^2^ = 0.021  Interaction effect  F= 0.17, 0.844;  η_p_^2^ = 0.001 |
| **Government vs.  Families** | 4.52 (0.30) | 4.77 (0.21) | 3.77 (0.34) | 4.87 (0.21) | 4.36 (0.51) | 4.52 (0.60) | Site effect  F=2.54, 0.112;  η_p_^2^ = 0.005  Rater effect  F=0.74, 0.478;  η_p_^2^ = 0.003  Interaction effect  F= 1.43, 0.240;  η_p_^2^ = 0.005 |
| **Families vs.  Persons with mental health problems** | 5.36 (0.23) | 3.42 (0.16) | 4.29 (0.26) | 2.98 (0.16) | 5.35 (0.38) | 4.19 (0.45) | **Site effect**  F= 37.16, **<0.001**;  η_p_^2^ = 0.067  **Rater effect**  F= 9.27, **<0.001**;  η_p_^2^ = 0.035  Interaction effect  F= 1.45, 0.236;  η_p_^2^ = 0.006 |

EMM = estimated marginal mean, SE = standard error.

Significant differences, p<0.05, are bold.

1. **Housing support**

|  | **PATIENTS** | | **FAMILIES** | | **CLINICIANS** | | **STATISTICS** |
| --- | --- | --- | --- | --- | --- | --- | --- |
|  | **Montreal** EMM (SE) | **Chennai**  EMM (SE) | **Montreal**  EMM (SE) | **Chennai**  EMM (SE) | **Montreal**  EMM (SE) | **Chennai**  EMM (SE) | **F, p, η_p_^2^** |
| **Government vs.  Persons with mental health problems** | 4.12 (0.29) | 4.98 (0.21) | 3.09 (0.33) | 3.86 (0.21) | 3.55 (0.49) | 3.81 (0.58) | **Site effect**  F= 4.19, **0.041**;  η_p_^2^ = 0.008  **Rater effect**  F= 8.78, **<0.001**;  η_p_^2^ = 0.033  Interaction effect  F= 0.26, 0.769;  η_p_^2^ = 0.001 |
| **Government vs.  Families** | 5.02 (0.31) | 6.08 (0.22) | 4.00 (0.35) | 5.13 (0.22) | 4.10 (0.53) | 5.24 (0.62) | **Site effect**  F= 11.37, **0.001**;  η_p_^2^ = 0.021  **Rater effect**  F= 6.71, **0.001**;  η_p_^2^ = 0.025  Interaction effect  F= 0.01, 0.990;  η_p_^2^ = 0.000 |
| **Families vs.  Persons with mental health problems** | 4.85 (0.24) | 4.00 (0.18) | 3.97 (0.28) | 3.18 (0.18) | 4.69 (0.42) | 4.00 (0.49) | **Site effect**  F= 8.90, **0.003**;  η_p_^2^ = 0.017  **Rater effect**  F= 7.69, **0.001**;  η_p_^2^ = 0.029  Interaction effect  F= 0.03, 0.974;  η_p_^2^ = 0.000 |

EMM = estimated marginal mean, SE = standard error.

Significant differences, p<0.05, are bold.

1. **Return to school/work**

|  | **PATIENTS** | | **FAMILIES** | | **CLINICIANS** | | **STATISTICS** |
| --- | --- | --- | --- | --- | --- | --- | --- |
|  | **Montreal** EMM (SE) | **Chennai**  EMM (SE) | **Montreal**  EMM (SE) | **Chennai**  EMM (SE) | **Montreal**  EMM (SE) | **Chennai**  EMM (SE) | **F, p, η_p_^2^** |
| **Government vs.  Persons with mental health problems** | 4.71 (0.29) | 4.64 (0.21) | 3.38 (0.34) | 3.72 (0.21) | 4.17 (0.50) | 4.95 (0.59) | Site effect  F= 1.27, 0.260;  η_p_^2^ = 0.002  **Rater effect**  F= 9.47, **<0.001**;  η_p_^2^ = 0.035  Interaction effect  F= 0.63, 0.531;  η_p_^2^ = 0.002 |
| **Government vs.  Families** | 4.88 (0.30) | 5.63 (0.21) | 4.20 (0.34) | 4.76 (0.21) | 4.38 (0.51) | 5.62 (0.60) | **Site effect**  F= 7 .23, **0.007**;  η_p_^2^ = 0.014  **Rater effect**  F= 4.14, **0.017**;  η_p_^2^ = 0.016  Interaction effect  F= 0.31, 0.736;  η_p_^2^ = 0.001 |
| **Families vs.  Persons with mental health problems** | 5.45 (0.25) | 4.37 (0.18) | 4.44 (0.28) | 3.56 (0.18) | 5.41 (0.42) | 4.76 (0.50) | **Site effect**  F= 10.79, **0.001**;  η_p_^2^ = 0.020  **Rater effect**  F= 9.57, <**0.001**;  η_p_^2^ = 0.035  Interaction effect  F= 0.21, 0.808;  η_p_^2^ = 0.001 |

EMM = estimated marginal mean, SE = standard error.

Significant differences, p<0.05, are bold.

1. **Mental health services cost**

|  | **PATIENTS** | | **FAMILIES** | | **CLINICIANS** | | **STATISTICS** |
| --- | --- | --- | --- | --- | --- | --- | --- |
|  | **Montreal** EMM (SE) | **Chennai**  EMM (SE) | **Montreal**  EMM (SE) | **Chennai**  EMM (SE) | **Montreal**  EMM (SE) | **Chennai**  EMM (SE) | **F, p, η_p_^2^** |
| **Government vs.  Persons with mental health problems** | 3.81 (0.26) | 4.01 (0.19) | 2.33 (0.30) | 3.12 (0.19) | 2.90 (0.44) | 3.38 (0.52) | Site effect  F= 3.12, 0.078;  η_p_^2^ = 0.006  **Rater effect**  F= 12.64, **<0.001**;  η_p_^2^ = 0.046  Interaction effect  F= 0.75, 0.472;  η_p_^2^ = 0.003 |
| **Government vs.  Families** | 3.99 (0.28) | 4.52 (0.20) | 2.92 (0.33) | 4.72 (0.20) | 3.03 (0.48) | 3.38 (0.57) | **Site effect**  F= 8.92, **0.003**;  η_p_^2^ = 0.017  **Rater effect**  F= 3.84, **0.022**;  η_p_^2^ = 0.015  **Interaction effect**  F= 3.51, **0.031**;  η_p_^2^ = 0.013 |
| **Families vs.  Persons with mental health problems** | 4.88 (0.22) | 3.70 (0.16) | 3.74 (0.26) | 3.17 (0.16) | 4.78 (0.39) | 4.14 (0.45) | **Site effect**  F= 10.93, **0.001**;  η_p_^2^ = 0.021  **Rater effect**  F= 9.74, **<0.001**;  η_p_^2^ = 0.036  Interaction effect  F= 1.24, 0.291;  η_p_^2^ = 0.005 |

EMM = estimated marginal mean, SE = standard error.

Significant differences, p<0.05, are bold.

1. **Medication cost**

|  | **PATIENTS** | | **FAMILIES** | | **CLINICIANS** | | **STATISTICS** |
| --- | --- | --- | --- | --- | --- | --- | --- |
|  | **Montreal** EMM (SE) | **Chennai**  EMM (SE) | **Montreal**  EMM (SE) | **Chennai**  EMM (SE) | **Montreal**  EMM (SE) | **Chennai**  EMM (SE) | **F, p, η_p_^2^** |
| **Government vs.  Persons with mental health problems** | 3.42 (0.25) | 3.75 (0.18) | 2.44 (0.29) | 3.04 (0.18) | 2.79 (0.43) | 3.24 (0.50) | Site effect  F=2.88, 0.090;  η_p_^2^ = 0.006  **Rater effect**  F= 7.07, **0.001**;  η_p_^2^ = 0.026  Interaction effect  F= 0.17, 0.844;  η_p_^2^ = 0.001 |
| **Government vs.  Families** | 3.71 (0.30) | 4.46 (0.21) | 2.94 (0.34) | 4.67 (0.21) | 2.79 (0.51) | 4.00 (0.60) | **Site effect**  F= 14.98, **<0.001**;  η_p_^2^ = 0.028  Rater effect  F=1.43, 0.240;  η_p_^2^ = 0.005  Interaction effect  F= 1.63, 0.197;  η_p_^2^ = 0.006 |
| **Families vs.  Persons with mental health problems** | 5.08 (0.24) | 3.90 (0.17) | 3.72 (0.28) | 3.29 (0.17) | 4.70 (0.42) | 3.76 (0.48) | **Site effect**  F= 10.97, **0.001**;  η_p_^2^ = 0.021  **Rater effect**  F= 10.29, **<0.001**;  η_p_^2^ = 0.038  Interaction effect  F= 1.49, 0.227;  η_p_^2^ = 0.006 |

EMM = estimated marginal mean, SE = standard error.

Significant differences, p<0.05, are bold.

1. **Substance use treatment cost**

|  | **PATIENTS** | | **FAMILIES** | | | **CLINICIANS** | | | **STATISTICS** |
| --- | --- | --- | --- | --- | --- | --- | --- | --- | --- |
|  | **Montreal** EMM (SE) | **Chennai**  EMM (SE) | | **Montreal**  EMM (SE) | **Chennai**  EMM (SE) | | **Montreal**  EMM (SE) | **Chennai**  EMM (SE) | **F, p, η_p_^2^** |
| **Government vs.  Persons with mental health problems** | 4.33 (0.34) | 6.32 (0.24) | | 2.56 (0.39) | 5.74 (0.24) | | 3.21 (0.58) | 4.24 (0.68) | **Site effect**  F= 33.06, **<0.001**;  η_p_^2^ = 0.060  **Rater effect**  F= 9.84, **<0.001**;  η_p_^2^ = 0.037  **Interaction effect**  F= 3.11, **0.046**;  η_p_^2^ = 0.012 |
| **Government vs.  Families** | 4.33 (0.32) | 7.03 (0.22) | | 3.02 (0.36) | 6.24 (0.22) | | 3.17 (0.53) | 5.10 (0.62) | **Site effect**  F= 62.03, **<0.001**;  η_p_^2^ = 0.107  **Rater effect**  F= 9.85, **<0.001**;  η_p_^2^ = 0.037  Interaction effect  F= 1.11, 0.331;  η_p_^2^ = 0.004 |
| **Families vs.  Persons with mental health problems** | 5.21 (0.33) | 6.40 (0.23) | | 4.07 (0.38) | 6.04 (0.23) | | 4.80 (0.57) | 5.76 (0.65) | **Site effect**  F= 15.40, <**0.001**;  η_p_^2^ = 0.029  **Rater effect**  F= 3.20, **0.041**;  η_p_^2^ = 0.012  Interaction effect  F= 1.05, 0.350;  η_p_^2^ = 0.004 |

EMM = estimated marginal mean, SE = standard error.

Significant differences, p<0.05, are bold.

1. **Stigma reduction**

|  | **PATIENTS** | | **FAMILIES** | | | **CLINICIANS** | | | **STATISTICS** |
| --- | --- | --- | --- | --- | --- | --- | --- | --- | --- |
|  | **Montreal** EMM (SE) | **Chennai**  EMM (SE) | | **Montreal**  EMM (SE) | **Chennai**  EMM (SE) | | **Montreal**  EMM (SE) | **Chennai**  EMM (SE) | **F, p, η_p_^2^** |
| **Government vs.  Persons with mental health problems** | 3.68 (0.28) | 3.27 (0.20) | | 2.56 (0.31) | 2.45 (0.20) | | 4.10 (0.47) | 4.00 (0.55) | Site effect  F=0.53, 0.468;  η_p_^2^ = 0.001 **Rater effect**  F= 11.15, **<0.001**;  η_p_^2^ = 0.041  Interaction effect  F= 0.20, 0.816;  η_p_^2^ = 0.001 |
| **Government vs.  Families** | 3.99 (0.32) | 4.18 (0.23) | | 3.43 (0.37) | 3. 29 (0.23) | | 4.62 (0.54) | 4.43 (0.63) | Site effect  F= 0.02, 0.890;  η_p_^2^ = 0.000  **Rater effect**  F= 4.67, **0.010**;  η_p_^2^ = 0.018  Interaction effect  F= 0.19, 0.824; η_p_^2^ = 0.001 |
| **Families vs.  Persons with mental health problems** | 5.38 (0.25) | 4.37 (0.18) | | 3.69 (0.29) | 3.34 (0.18) | | 4.93 (0.42) | 3.67 (0.49) | **Site effect**  F= 11.16, **0.001**;  η_p_^2^ = 0.021  **Rater effect**  F= 18.07, **<0.001**;  η_p_^2^ = 0.065  **Interaction effect**  F= 1.39, 0.249; η_p_^2^ = 0.005 |

EMM = estimated marginal mean, SE = standard error.

Significant differences, p<0.05, are bold.
