# Supplementary material for: Whose responsibility? Part 2 of 2: views of patients, families, and clinicians about responsibilities for addressing the needs of persons with mental health problems in Chennai, India and Montreal, Canada
Source: Int J Ment Health Syst. 2022 Jan 10;16:2. doi: 10.1186/s13033-021-00511-w (PMC8744303; doi:10.1186/s13033-021-00511-w)
Supplement: Supplementary file 2 — Additional file 2. Post-hoc tests for significant differences between raters (refer to additional file 1). [file 13033_2021_511_MOESM2_ESM.docx]

**Additional file 2. Post-hoc tests for significant differences between raters** (See Additional file 1)

| **Rater** | | **Government vs.**  **Persons with mental health problems** | | | | | | | | | | | |
| --- | --- | --- | --- | --- | --- | --- | --- | --- | --- | --- | --- | --- | --- |
|  |  | **Financial** | **Housing** | | | **School/ work** | **MH services** | **Medications** | | | **Alcohol/ Drugs** | **Stigma** | |
| Patient | Family | **0.045 (P<F)** | **<0.001 (P<F)** | | | **<0.001 (P<F)** | **<0.001 (P<F)** | **0.001 (P<F)** | | | **<0.001 (P<F)** | **<0.001 (P<F)** | |
| Patient | Clinician | 0.431 | 0.114 | | | 1.000 | 0.126 | 0.352 | | | **0.003 (P<C)** | 0.440 | |
| Family | Clinician | **0.010 (C<F)** | 1.000 | | | 0.057 | 0.841 | 1.000 | | | 1.000 | **<0.001 (C<F)** | |
|  |  | **Government vs. Families** | | | | | | | | | | | |
| Patient | Family | NA | | **0.001 (P<F)** | **0.013 (P<F)** | | 0.268 | | NA | **0.001 (P<F)** | | | **0.038 (P<F)** |
| Patient | Clinician | NA | | 0.147 | 1.000 | | **0.031 (P<C)** | | NA | **0.002 (P<C)** | | | 1.000 |
| Family | Clinician | NA | | 1.000 | 0.716 | | 0.419 | | NA | 0.851 | | | **0.038 (C<F)** |
|  |  | **Families vs. Persons with mental health problems** | | | | | | | | | | | |
| Patient | Family | **0.001 (P<F)** | | **<0.001 (P<F)** | **<0.001 (P<F)** | | **<0.001 (P<F)** | | **<0.001 (P<F)** | **0.038 (P<F)** | | | **<0.001 (P<F)** |
| Patient | Clinician | 0.742 | | 1.000 | 1.000 | | 1.000 | | 1.000 | 0.831 | | | 0.320 |
| Family | Clinician | **0.002 (C<F)** | | 0.097 | **0.009 (C<F)** | | **0.008 (C<F)** | | 0.127 | 1.000 | | | 0.096 |

MH = mental health, P = patient, F = family, C = clinician.

NA = non-applicable since simple main effect is not significant.

Significant differences, p<0.05 (Bonferroni adjusted for multiple comparisons), are bold.

Parentheses indicate the direction of the difference between rater pairs (e.g., P<F, means that patient raters assigned less responsibility than family raters). Every pair was mentioned once to avoid redundancy.
