# Supplementary material for: Whose responsibility? Part 2 of 2: views of patients, families, and clinicians about responsibilities for addressing the needs of persons with mental health problems in Chennai, India and Montreal, Canada
Source: Int J Ment Health Syst. 2022 Jan 10;16:2. doi: 10.1186/s13033-021-00511-w (PMC8744303; doi:10.1186/s13033-021-00511-w)
Supplement: Supplementary file 3 — Additional file 3. Post-hoc tests for significant differences between support needs in Montreal and Chennai (refer to Fig. 3). [file 13033_2021_511_MOESM3_ESM.docx]

**Additional file 3. Post-hoc tests for significant differences between support needs in Montreal and Chennai** (see Fig. 3)

| **Dependent variable** | **Support need** | | **Site** | |
| --- | --- | --- | --- | --- |
|  |  |  | **Montreal (p)** | **Chennai (p)** |
| Mean responsibility assigned to Government vs. Persons with mental health problems | Financial (Fi) | Housing | **0.003 (F>H)** | **<0.001 (Fi>H)** |
|  |  | School/work | 0.239 | **<0.001 (Fi>S/W)** |
|  |  | Mental health services | 0.588 | 0.439 |
|  |  | Medication | 0.200 | 0.944 |
|  |  | Alcohol/Drugs | 1.000 | **<0.001 (Fi>A/D)** |
|  |  | Stigma | 0.984 | 0.737 |
|  | Housing (H) | Financial | **0.003** | **<0.001** |
|  |  | School/work | 0.814 | **0.013 (H<S/W)** |
|  |  | Mental health services | **<0.001 (H<MHS)** | **<0.001 (H<MHS)** |
|  |  | Medication | **<0.001 (H<Med)** | **<0.001 (H<Med)** |
|  |  | Alcohol/Drugs | **0.003 (H<A/D)** | **0.003 (H>A/D)** |
|  |  | Stigma | **<0.001 (H<S)** | **<0.001 (H<S)** |
|  | School/Work (S/W) | Financial | 0.239 | **<0.001** |
|  |  | Housing | 0.814 | **0.013** |
|  |  | Mental health services | **0.002 (S/W<MHS)** | **0.015** |
|  |  | Medication | **<0.001 (S/W<Med)** | **<0.001 (S/W<Med)** |
|  |  | Alcohol/Drugs | 0.195 | **<0.001 (S/W>A/D)** |
|  |  | Stigma | **0.050 (S/W<S)** | **<0.001 (S/W<S)** |
|  | Mental health services (MHS) | Financial | 0.588 | 0.439 |
|  |  | Housing | **<0.001** | **<0.001** |
|  |  | School/work | **0.002** | **0.015** |
|  |  | Medication | 0.995 | 0.966 |
|  |  | Alcohol/Drugs | 0.763 | **<0.001 (MHS>A/D)** |
|  |  | Stigma | 0.980 | **0.019 (MHS<S)** |
|  | Medication (Med) | Financial | 0.200 | 0.944 |
|  |  | Housing | **<0.001** | **<0.001** |
|  |  | School/work | **<0.001** | **<0.001** |
|  |  | Mental health services | 0.995 | 0.966 |
|  |  | Alcohol/Drugs | 0.355 | **<0.001 (Med>A/D)** |
|  |  | Stigma | 0.768 | 0.177 |
|  | Alcohol/Drugs (A/D) | Financial | 1.000 | **<0.001** |
|  |  | Housing | **0.003** | **0.003** |
|  |  | School/work | 0.195 | **<0.001** |
|  |  | Mental health services | 0.763 | **<0.001** |
|  |  | Medication | 0.355 | **<0.001** |
|  |  | Stigma | 0.997 | **<0.001 (A/D<S)** |
|  | Stigma (S) | Financial | 0.984 | 0.737 |
|  |  | Housing | **<0.001** | **<0.001** |
|  |  | School/work | **0.050** | **<0.001** |
|  |  | Mental health services | 0.980 | **0.019** |
|  |  | Medication | 0.768 | 0.177 |
|  |  | Alcohol/Drugs | 0.997 | **<0.001** |

Bold is significant. Parentheses indicate the need area where government was assigned more responsibility (e.g., Fi>H, means that government was seen as more responsible for financial support needs than for housing needs). Every pair was mentioned once to avoid redundancy.

Patients’, families’ and clinicians’ ratings were combined.
